# Impact of warm-up methods on strength-speed for sprinters in athletics: a mini review

**DOI:** 10.3389/fspor.2024.1360414

**Published:** 2024-02-27

**Authors:** Eduardo Herrera, Cesar Osorio-Fuentealba

**Affiliations:** ^1^ Programa de Magíster en Ciencias Aplicadas al Movimiento y la Cognición Humana, Departamento de Kinesiología, Universidad Metropolitana de Ciencias de la Educación, Santiago, Chile; ^2^ Núcleo de Bienestar y Desarrollo Humano (NUBIDEH), Centro de Investigación en Educación (CIE-UMCE), Universidad Metropolitana de Ciencias de la Educación, Santiago, Chile

**Keywords:** warm-up, skeletal muscle, power, flexibility, sprint, athletes

## Abstract

In athletics, achieving peak performance during competitions is crucial. Warm-up strategies play a crucial role in optimizing the strength-speed performance of sprinters in athletics, especially tailored to the physiological demands of speed events. The need to balance flexibility, prevent injuries, and enhance power output makes the selection of an effective warm-up protocol essential. This narrative review examines different warm-up methods used by athletes and their effects on strength-speed in sprinters in athletics. The main findings indicate that Foam Rolling (FR), Isometric Exercises and Pre-Competitive Massages have no significant effects on sprint performance. Static stretching and prolonged Pre-Competitive Massages have negative impacts on strength and power. The Vibration Platform enhances step length, step rate and running velocity, jump height and total number of jumps performed in a 30-s period in non-experienced sprinters. Eccentric Exercise increases vertical force, Post-Activation Potentiation (PAP) demonstrates a reduction in 100-meter time and short-term improvement in vertical and horizontal jumps. Blood Flow Restriction (BFR) significantly improving jump height and flight time. Various warm-up methods have been identified, some focusing on flexibility, others potentially detrimental, and some enhancing strength and power. Implementing effective warm-ups, particularly those promoting strength and power, poses a challenge for coaches seeking reliable alternatives to boost performance.

## 1 Introduction

Warming up is a procedure that is used in mostly all sports with the intention to gradually adapt the body physically and mentally for the main activity afterwards, preparing athletes for subsequent stress to enhance this activity performance (1–4), and to reduce the risk of injuries during that activity (5, 6). Although the positive effects of warm-up have been well-documented, negative effects have also been noted. Long warm-ups have been reported to cause fatigue and may detract from performance (5, 7).

Common warm-ups include cardio activities like calisthenics, dynamic stretching, or sport-specific movements, in athletics, particularly speed events, jumps, and throws, involve explosive efforts lasting a few seconds (8). Optimal results in these disciplines require strength and speed, making it essential to maximize power for enhanced sports performance (9). Adequate preparation, with a crucial emphasis on warm-up, aims to ready athletes for competition and acutely enhance sports performance (10). An effective warm-up is known to improve athletes' physical performance and reduce the risk of sports injuries (11). However, the challenge lies in the variety of warm-up methods, as not all align with the physiological demands of the task and may have negative effects on sports performance (12). Therefore, identifying the most optimal warm-up for athletics is critical.

This work aims to discuss the types of warm-ups used in athletics and their impact on the strength-speed or power variable to maximize competition performance, providing a comprehensive overview of the current state of the art regarding the effect of warm-up on athlete performance.

## 2 Types of warm-ups used in athletics

Based on a literature review and observations from national and international championships, professional training sessions, and athlete warm-ups, the following warm-up methods can be summarized:

### 2.1 Foam rolling

Warm-up method in which athletes use their bodyweight to apply rolling pressure to the soft tissues to the target muscles (13). Foam rolling (FR) is a form of self-massage in which the targeted musculature is rolled and compressed utilizing a FR device (14).

Consequently, FR can be considered a form of self-induced massage because the pressure that the roller exerts on the muscles resembles the pressure exerted on the muscles through manual manipulation by the user himself (15).

• Increases range of motion without negatively affecting subsequent performance (16).
• Optimal implementation remains uncertain, posing challenges for coaches (17).
• No significant decrease in sprint time observed in relation to speed races (18).

### 2.2 Static stretching

Static stretching involves maintaining constant muscle tension at the end of the passive range of the muscle involved (19).

• Potentially positive effects on flexibility and prevention of muscle-tendon injuries. Evidence suggests negative impacts on strength and power, affecting subsequent performance (20).
• Adversely affects short distance races (21, 22) and shorter race times in 100 meters (23).

### 2.3 Dynamic stretching

Dynamic stretching has been defined as a controlled movement through the active range of motion for each joint (24).

• Reduces passive muscle stiffness, increases range of motion, and aids injury prevention (25).
• Enhances muscular performance, specifically in leg extension power (26) and drecreasing joint and vertical stiffness, improving the overall running economy (27).
• Acute changes in strength, power, and balance are less clear (28).

### 2.4 Pre-Competitive massages

This warm up method is based on the mechanical manipulation of body tissues, applying pressure in a rhythmic manner (29).

• No positive effects on maximum strength, jumps, balance, and agility.
• Negative effects on lower limb maximum strength, vertical jump, and sprints with prolonged massages (30).
• Questionable use in speed races due to demonstrated lower performance (31, 32).

### 2.5 Vibration platform

In this warm-up, the athlete must stand on a platform that generates vertical sinusoidal vibration at frequencies between 25 and 50 Hz, transmitting these mechanical stimuli throughout the body, stimulating the sensory receptors, most likely muscle spindles (33). Causing activation of the alpha-motoneurons and initiates muscle contractions comparable to the “tonic vibration reflex” (33)

• Safe method, seemingly with no injury incidents and increases flexibility and subjects' range of motion (34).
• Improve kinematical characteristics of sprint running as step length, step rate and running velocity and explosive strength characteristics as jump height, total number of jumps performed in a period of 30 s in non-experienced sprinters (33)
• Efficacy of body vibration as an ergogenic aid in 30-meter sprints is questioned (35).

### 2.6 Isometric exercises

This warm-up method involves contraction of the muscles without performing any external movement (36).

• Demonstrated improvements in sports like baseball (37) and rowing (38).
• Isometric warm-up had no significant effects on sprint performance and counter-movement jump height (39).

### 2.7 Eccentric exercise

This warm-up method is characterized by the lengthening of the muscle-tendon complex and occurs when a force applied to the muscle exceeds the momentary force of the muscle itself (40), resulting in the forced lengthening of the muscle-tendon system while contracting (41).

• Increases vertical force, resulting in higher speed at the activity's onset (42).
• Significant improvements observed in counter-movement jumps and 30-meter sprints (43).
• Enhances speed performance or in rebound activities such as jump (44, 45)

### 2.8 Jumping warm-Up

This warm-up method can be described by the stretch-shortening cycle, where you go from a rapid eccentric muscle contraction to a rapid concentric muscle contraction (46), is characterized by the operation of the stretch-shortening cycle (SSC) that develops during the transition from a rapid eccentric muscle contraction (deceleration or a negative phase) to a rapid concentric muscle contraction (acceleration or a positive phase) (46).

• Plyometric activations show significant increases in subsequent jump height and maximum power (47).
• Adding plyometric exercises during warm-up may improve performance in short-distance races (48, 49).

### 2.9 Post-Activation potentiation (PAP)

This warm-up method involves maximal or submaximal stimuli that provide a window of improvement in muscle power (50), this improvement depends on the balance of fatigue and potentiation, which in turn depends on the type of exercise, volume, intensity and recovery time (51). Originally defined by Robbins (52), PAP is a phenomenon by which the force exerted by a muscle is increased due to its previous contraction. Post-activation potentiation is a theory that purports that the contractile history of a muscle influences the mechanical performance of subsequent muscle contractions.

• Studied improvements in shot put distance in competitive throwers (53), reduced 100-meter time with submaximal squats (54), and short-term enhancement of vertical (55) and horizontal jumps (56).

### 2.10 Blood flow restriction (BFR)

This warm-up method is based on the use of pressurized cuffs in the proximal portion of the muscle of each extremity, whether upper or lower, this pressure guarantees arterial supply and prevents venous return from the corresponding area (57).

• Successfully employed in some studies, significantly improving jump height and flight time (58).
• observed in various studies (59, 60).

In conclusion, understanding the nuanced effects of different warm-up methods is crucial for coaches and athletes seeking to optimize performance in athletics competitions, particularly in speed events.

## 3 Discussion

Initially, we encountered warm-ups focused on the acute increase in flexibility. While flexibility plays a crucial role in preventing musculoskeletal injuries (11), prolonged flexibility-focused warm-ups may contribute to injuries and a decline in sports performance (61). It has even been demonstrated that a single session of static stretching can significantly reduce maximum strength and power (62). However, if a choice must be made between static and dynamic stretching, dynamic stretching presents greater benefits compared to static stretching and no stretching at all (63). Similar outcomes are obtained with massages as a warm-up method, as they do not directly enhance performance (64) and may only influence flexibility without impacting motor capabilities (65), potentially being detrimental if excessively long. The same caution should be applied to dynamic stretching and foam roller use if done excessively (66). In the same way, the use of foam rolling as a warm-up activity (i.e., pre-rolling) is still in question, some evidence indicate that pre-rolling causes a small acute improvement in sprint performance and flexibility, while its effect on jump and strength performance was negligible (17).

Interestingly the effects of pre-rolling on sprint performance seem to be more relevant for elite athletes, while it is possible that recreationally active individuals may not benefit substantially from pre-rolling (17).

Vibration platform exercises and isometric exercises do not seem to significantly influence strength, as indicated in Table 1. Some authors argue against the performance-enhancing effects of a vibration platform protocol (68), and isometric warm-up shows similar results in speed compared to a dynamic stretching protocol (69).

**Table 1:** Effects of different types of warm-up on flexibility, strength and power.

Author	Type of Warm-Up	Effects	Applicable in competition
Wiewelhove et al. (17)	Foam rolling	↑ Flexibility, does not influence the strength.	Yes
Chaabene et al. (20)	Static Stretching	↑ Flexibility, negatively influences strength.	Yes
Behara and Jacobson (67)	Dynamic Stretching	↑ Flexibility, does not influence the strength.	No
Mine, Lei and Nakayama (30)	Pre-Competitive Massages	Negatively influences strength.	Yes
Bullock et al. (35)	Vibration Platform	Does not influence the strength.	No
Ullman, Fernandez and Klein (39)	Isometric Exercise	Does not influence the strength.	Yes
Cuenca-Fernández et al. (42)	Eccentric Exercise	Positively influences the strength.	No
Tobin and Delahunt (47)	Jumping Warm-Up	↑ Jump height and maximum power.	Yes
Linder et al. (54)	Post-Activation Potentiation	Reduces track-sprint times	No
Doma et al. (59)	Blood Flow Restriction	↑ Jump height, flight time and power.	Yes

Regarding jumping warm-up, an improvement in strength and power is observed, but contrary to Creekmur's statement, other authors have concluded that it does not impact subsequent running performance (70). More research is necessary, in order to clarify the effects of jumping warm-up on running performance.

Eccentric exercises and Post-Activation Potentiation result in an acute performance increase, even reflected in subsequent races (71). However, implementing these methods in athletics competitions poses challenges due to time constraints and equipment logistics dictated by athletics regulations (72). Additionally, the neuromuscular effects of eccentric exercises, including a potential decrease in maximum strength and force development rate, must be considered (73).

Blood flow restriction (BFR) warm-up, as mentioned earlier, shows gains in power and jump height. Some authors suggest that working with BFR reduces fatigue, providing a longer interval to benefit from post-activation potentiation (60). However, more information is needed to determine its impact on improving running times.

Finally, the warm-ups reported in this review are based on what has been observed in national and international championships, as well as information gathered from the literature. The most used warm-ups include two types of stretching, static and dynamic stretching, the use of a foam roller, and to a lesser extent, jumps. Other warm-up techniques are challenging to implement in competition settings as they require greater equipment or weights, which would be prohibited in the athletics warm-up area due to safety reasons.

### 3.1 Limitations

As this is a narrative review, the authors attempted to reflect the essential state of the literature by performing an extended study search. However, because there is a vast number of studies, especially regarding the effects of stretching on flexibility in humans, it was necessary to focus the literature search, which possibly led to some studies missing in the review article. To analyze studies addressing our research question, we started by screening recent systematic review articles addressing the topic (17, 30, 39, 50, 58, 65). Subsequently, related articles and reference lists were screened to find articles excluded in the aforementioned systematic reviews. Furthermore, only studies that investigated the effects of warm-up on strength or strength-related parameters, such as maximal torque, maximal voluntary contractions (eccentric, isometric, or concentric), and muscle power, were considered in this review.

## 4 Conclusion

Warm-ups should be individualized and sport specific. An inappropriate warm-up could be detrimental, negatively affecting strength. In the case of athletics, the warm-up should focus on increasing the subjects' power. While effective protocols exist, their applicability is limited. Therefore, working with blood flow restriction could be a beneficial tool, but further research is required to understand its real effects and whether it enhances competition performance.
