# Supplementary figures and images for: Impact of warm-up methods on strength-speed for sprinters in athletics: a mini review
Source: Front Sports Act Living. 2024 Feb 27;6:1360414. doi: 10.3389/fspor.2024.1360414 (PMC10927743; doi:10.3389/fspor.2024.1360414)

Supplementary figure

Selection of studies

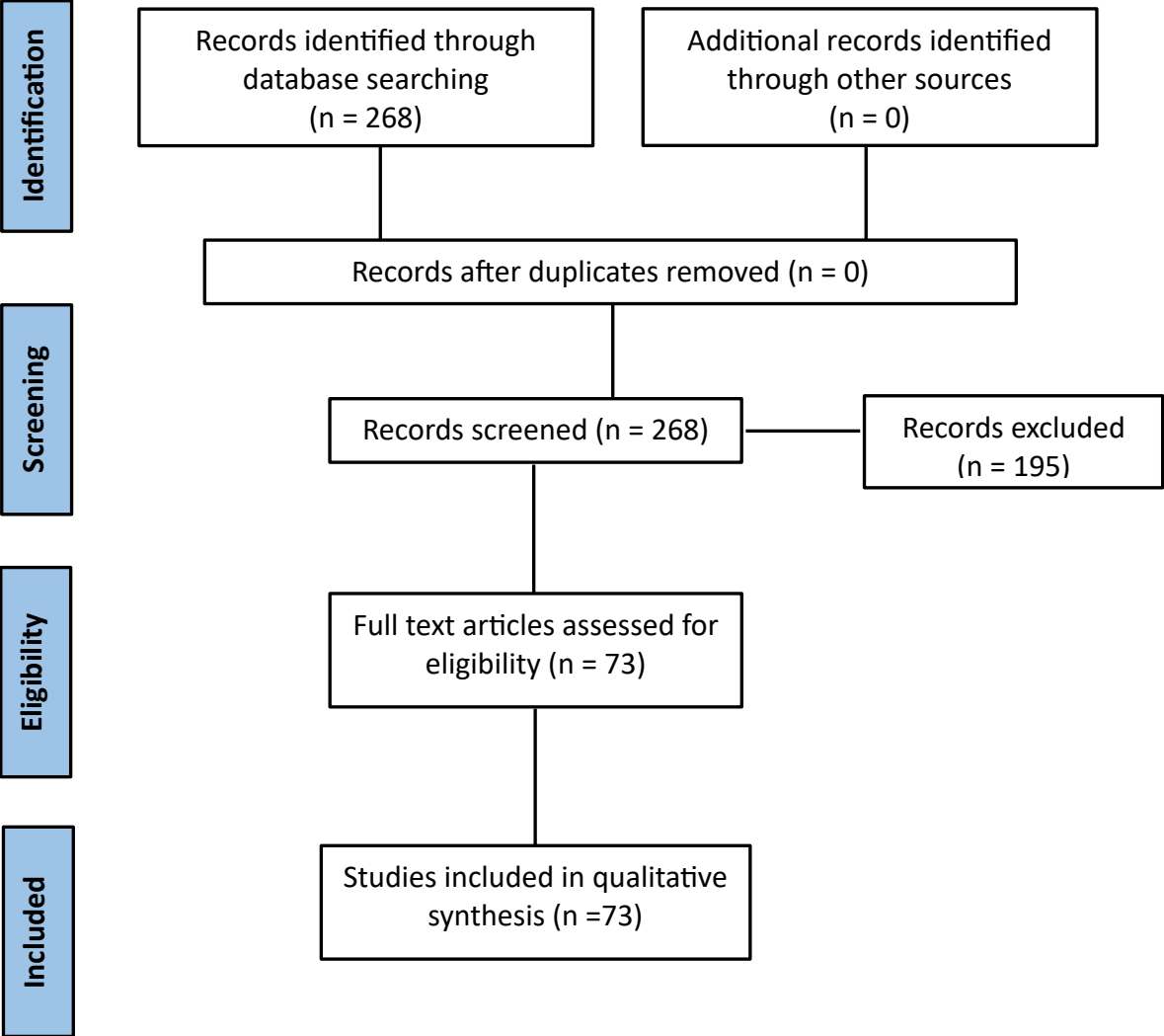

Supplement: Supplementary file 1 [file Image1.pdf]
